# Overlap of Sjögren's Syndrome and Immune-Mediated Necrotizing Myopathy With Interstitial Lung Disease: A Case Report

**DOI:** 10.7759/cureus.70385

**Published:** 2024-09-28

**Authors:** Keiko Tanimura, Satoshi Omura, Masashi Egami, Rie Watanabe, Takayuki Nakano

**Affiliations:** 1 Department of Respiratory Medicine, Fukuchiyama City Hospital, Kyoto, JPN; 2 Department of Inflammation and Immunology, Graduate School of Medical Science, Kyoto Prefectural University of Medicine, Kyoto, JPN

**Keywords:** anti-srp antibody, immune mediated necrotizing myopathy (imnm), interstitial lung disease, non-specific interstitial lung disease (nsip), sjögren's syndrome

## Abstract

Immune-mediated necrotizing myopathy (IMNM), an inflammatory muscle disease, typically presents as severe muscle weakness due to immunologic mechanisms. Some cases also show cutaneous manifestations, interstitial lung disease, and sicca symptoms. In this report, we present a unique case of an elderly man with a history of mild and stable Sjögren's syndrome (SS) for over 10 years, who later developed interstitial lung disease and myositis, leading to a diagnosis of IMNM confirmed by the presence of anti-signal recognition particle antibodies. The coexistence of IMNM and SS is a rare occurrence, underscoring the need for our expertise and a comprehensive understanding of the unique characteristics of each disease for accurate diagnosis and effective treatment.

## Introduction

Immune-mediated necrotizing myopathy (IMNM) is an inflammatory myopathy characterized by severe muscle weakness, marked elevation of creatine kinase (CK), and progressive dysphagia. Pathologically, it is defined by the necrosis and regeneration of muscle fibers, with minimal inflammatory cell infiltration into muscle tissue [1]. Activation of the classical complement pathway plays a crucial role in the pathogenesis of this disease. IMNM is categorized into three subtypes based on serological findings: anti-signal recognition particle (SRP) antibody-positive, anti-3-hydroxy-3-methylglutaryl-coenzyme A reductase (HMGCR) antibody-positive, and seronegative types. Anti-SRP antibody-positive IMNM accounts for 5-17% of idiopathic inflammatory myopathies [2]. The onset usually occurs between the ages of 40 and 60 and is more common in women [3]. Here, we report the case of an elderly man with over a decade of stable Sjögren's syndrome (SS), who later developed anti-SRP-positive IMNM, accompanied by interstitial lung disease (ILD). The co-occurrence of anti-SRP-positive IMNM and SS is quite rare, representing only 0.51% of all IMNM cases [4]. Although previous reports suggest that IMNM cases overlapping with SS tend to present with relatively mild muscle symptoms and slow to moderate progression, this case demonstrated subacute disease progression and resistance to glucocorticoid and immunosuppressive therapy.

## Case presentation

**Figure 1:**
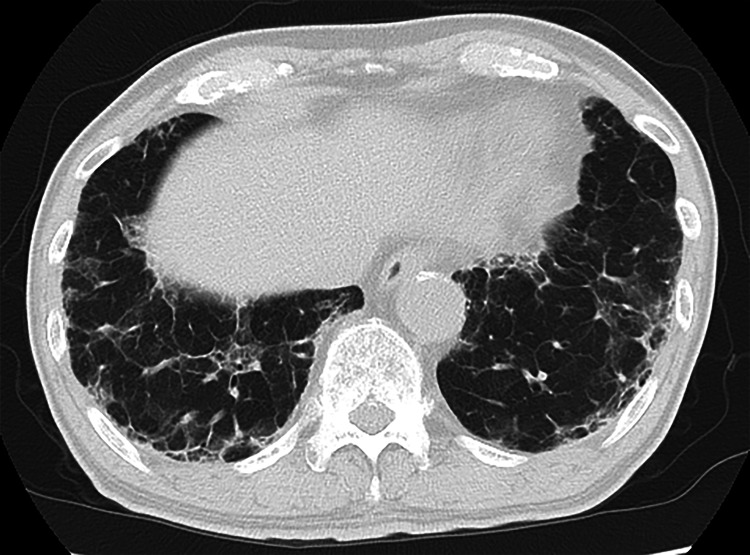
High-resolution lung CT scans show bilateral subpleural and peribronchovascular ground-glass opacities and reticular lesions with traction bronchiectasis, suggesting an NSIP pattern. NSIP: Non-specific interstitial pneumonia

A 75-year-old man diagnosed with SS 11 years ago through a minor salivary gland biopsy had been followed without treatment, as he exhibited only mild sicca symptoms and no other organ-specific or systemic manifestations beyond the exocrine glands. He presented with fever and dyspnea, and a chest CT scan (Figure 1) revealed interstitial lung disease with a nonspecific interstitial pneumonia (NSIP) pattern.

Bronchoalveolar lavage showed an elevated lymphocyte fraction (total cell count 0.7 × 10^5^/mL, with 59% lymphocytes, 12% neutrophils, 2% eosinophils, and 27% other cells, including macrophages). A transbronchial lung biopsy from the right lower lobe revealed pulmonary interstitial fibrosis with minimal inflammatory cell infiltration into the lung parenchyma and peripheral bronchi, consistent with fibrotic NSIP.

**Figure 2:**
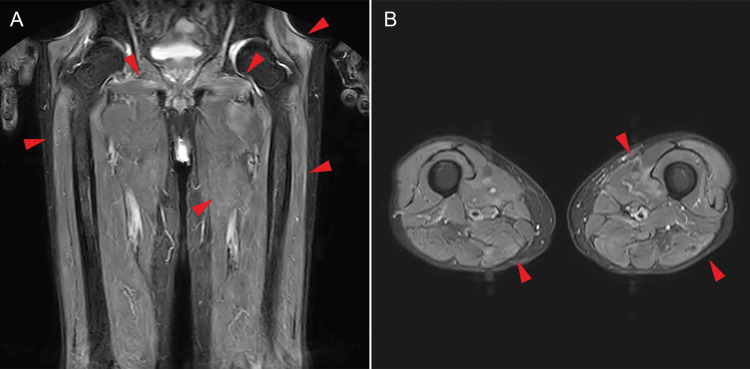
Fat-suppressed T2WI/STIR in the proximal muscles of the lower extremities. A - coronal, B - axial, T2WI - T2-weighted image, STIR - short tau inversion recovery Red arrowheads indicate high signals in the skeletal muscle, suggesting non-specific edema.

Over time, the patient developed worsening proximal muscle weakness, recurrent fever, dyspnea, and elevated CK levels. Magnetic resonance imaging (MRI) of the thighs (Figure 2) demonstrated high-signal intensity on short tau inversion recovery (STIR) and fat-suppressed T2-weighted images (T2WI) in the proximal muscles of the lower extremities, consistent with IMNM.

The serological analysis confirmed the presence of anti-SRP antibodies and the previously known anti-SS-A and anti-SS-B antibodies, leading to a diagnosis of anti-SRP-positive IMNM. At diagnosis, the patient's muscle weakness was rapidly progressing, and treatment with glucocorticoid and the immunosuppressive agent tacrolimus was initiated despite the absence of a muscle biopsy.

After the start of treatment, CK levels decreased, and the progression of the lung lesions stabilized. However, the patient's muscle weakness continued to worsen. On the 16th day of hospitalization, the patient suffered cardiopulmonary arrest due to the rupture of a coexisting abdominal aortic aneurysm and unfortunately passed away.

## Discussion

Recent European Neuromuscular Centre recommendations suggest that a diagnostic muscle biopsy is unnecessary when clinical symptoms of IMNM are present, along with positive anti-SRP or anti-HMGCR antibodies [1]. Instead, early initiation of potent immunosuppressive therapy is associated with a favorable prognosis [3]. However, patients with anti-SRP antibody-positive IMNM often develop severe muscle involvement early in the disease course and experience poor functional recovery despite treatment.

SS, an autoimmune disease characterized by lymphocytic infiltration of exocrine glands, such as the salivary and lacrimal glands, is frequently associated with muscle involvement. A multicenter cohort study of SS reported that 2.24% of patients presented with muscle weakness, including overlap cases with polymyositis, dermatomyositis, or inclusion body myositis, confirmed through muscle biopsy [5]. Most cases of myositis associated with SS present with relatively mild symptoms and show a better response to glucocorticoid and immunosuppressive therapy [6]. Although the study included a limited number of cases, anti-SRP IMNM overlap with SS showed better treatment outcomes compared to anti-SRP IMNM alone [4]. Conversely, muscle biopsies of anti-SRP IMNM-SS overlap cases revealed higher levels of inflammatory cell infiltration and membrane attack complex (MAC) deposition in the sarcolemma, indicating severe tissue injury mediated by activation of the classical complement pathway. On the other hand, myoblast fusion defects associated with decreased cytokine production impair muscle regeneration in patients with anti-SRP IMNM. However, SS-overlap cases demonstrated higher cytokine levels compared to IMNM-alone cases, which may explain the milder muscle manifestations observed in these patients [7].

In our case of anti-SRP IMNM-SS overlap, the patient presented with subacute muscle weakness and a poor initial response to glucocorticoid and immunosuppressive agents, which is typical for IMNM but differs somewhat from previous reports of SS overlap cases. The interval between the onset of IMNM and SS was more prolonged than in other reports, suggesting that SS, which had been stable for a prolonged period, may have had a marginal impact on the development of IMNM.

IMNM often presents with pulmonary involvement, and the prevalence varies according to serologic subtypes. The reported prevalence of ILD in anti-SRP antibody-positive IMNM ranges from 10% to 45%, higher than in anti-HMGCR antibody-positive or seronegative cases [8-12]. The most common ILD pattern observed in anti-SRP IMNM is NSIP in 63% of cases, followed by organizing pneumonia (OP) and lymphoid interstitial pneumonia (LIP), with most cases being asymptomatic [9]. Similarly, the prevalence of pulmonary involvement in SS is 10-20%, and the NSIP pattern is the most common, mirroring findings in IMNM-ILD cases [13,14]. Additionally, anti-SRP IMNM overlap with SS shows a higher prevalence of ILD compared to anti-SRP IMNM alone [4].

While radiographic and histopathologic evaluations are essential for diagnosing ILD, lung biopsy is often impractical in clinical practice due to the risk of respiratory failure. Therefore, fractional analysis of bronchoalveolar lavage fluid (BALF) is typically used to assess the pathophysiology of ILD. Although no studies directly compare IMNM and SS regarding BALF cellular composition, previous reports indicate that the median lymphocyte fraction exceeds 20% in SS and is approximately 9% in inflammatory muscle diseases [15,16]. In our case, the lymphocyte fraction in BALF was elevated, suggesting that increased infiltration of inflammatory cells may contribute to the development of both muscular and extra-muscular symptoms in SS-overlap IMNM compared to IMNM alone. However, the pathogenesis remains unclear due to the limited number of reports on these rare overlap cases. We anticipate that accumulating clinical cases will help advance therapeutic strategies.

## Conclusions

We report a case of SS complicated by progressive ILD and anti-SRP antibody-positive IMNM. In cases where multiple autoimmune diseases overlap, accurate diagnosis and early therapeutic intervention based on symptom progression are essential for improving treatment outcomes and functional prognosis.
